# “It doesn’t matter if you are in charge of the trees, you always miss the trees for the forest”: Power and the illusion of explanatory depth

**DOI:** 10.1371/journal.pone.0297850

**Published:** 2024-04-16

**Authors:** Robert Körner, Astrid Schütz, Lars-Eric Petersen

**Affiliations:** 1 Department of Psychology, Martin-Luther-University of Halle-Wittenberg, Halle, Germany; 2 Department of Psychology, Otto-Friedrich-University of Bamberg, Bamberg, Germany; Li Ka Shing Faculty of Medicine, The University of Hong Kong, HONG KONG

## Abstract

Power can increase overconfidence and illusory thinking. We investigated whether power is also related to the illusion of explanatory depth (IOED), people’s tendency to think they understand the world in more detail, coherence, and depth than they actually do. Abstract thinking was reported as a reason for the IOED, and according to the social distance theory of power, power increases abstract thinking. We linked these literatures and tested construal style as a mediator. Further, predispositions can moderate effects of power and we considered narcissism as a candidate because narcissism leads to overconfidence and may thus increase the IOED especially in combination with high power. In three preregistered studies (total *N* = 607), we manipulated power or measured feelings of power. We found evidence for the IOED (regarding explanatory knowledge about devices). Power led to general overconfidence but had only a small impact on the IOED. Power and narcissism had a small interactive effect on the IOED. Meta-analytical techniques suggest that previous findings on the construal-style-IOED link show only weak evidential value. Implications refer to research on management, power, and overconfidence.

## Introduction

Power holders’ thinking and decision-making typically have a large impact on organizations and society. Thus, it is important to understand whether power holders are susceptible to cognitive biases and under which conditions their assessments may be flawed. For example, as president of Brazil, Jair Bolsonaro is at the top of a social hierarchy and has extensive power. When the COVID-19 pandemic was increasingly spreading around the world in 2020 and top physicians and epidemiologists urged caution about the risk of contagion, Bolsonaro called the coronavirus a flu and did not do much to bolster Brazil’s health system or protect its citizens. Consequently, Brazil was among the countries worst affected by COVID-19 in 2020 [1]. If Bolsonaro had been asked how accurate and comprehensive his understanding of the virus and its consequences was, he may have been very confident about the depth of his knowledge—a reaction that could be considered typical of narcissists [2,3] but may also be observed in others: When people who are confident about their knowledge are asked to explain specifically how something works, they often have to admit that their knowledge is lower than they thought before they had to provide such a detailed explanation. This phenomenon is called the illusion of explanatory depth (IOED; [4]). Here, we investigated the impact of power on the IOED.

### Power and illusions

At a general level, power is understood as social influence [5] and is grounded in asymmetric control over valued resources [6]. Typically, researchers distinguish between structural power and a sense of power. Structural power describes whether people actually possess power—a concept that is also called objective power. In organizational research people who have structural power and thus objective control over valued resources are often referred to as power holders [7]. To manipulate structural power in experiments, researchers have often used role assignment tasks (e.g., manager-subordinate scenarios; [8]). However, most psychological research conceives of power as a psychological property: an individual’s perception of their ability to potentially influence others (their sense of power; [5]). Moreover, power has been conceptualized as a stable trait or as a situation-specific state [5,8,9]. The results from studies on power as a trait (e.g., with a trait measure of sense of power) typically converge with results from studies that measure power as a state (e.g., manipulated through episodic recall; [10–12]). Even if sense of power is different from structural power [13] the two forms are typically highly correlated [5]. In that vein, Magee and Galinsky [14], in their seminal review on power, assume that research based on power manipulations targeting sense of power are also applicable to structural power, and that it can therefore be assumed that results from studies on sense of power are also applicable to organizational contexts [15–17].

Whereas power can have several positive consequences such as optimism, positive emotion, and approach-related behavior [10], it has also been reported to lead to illusory thinking. For example, research has shown that power is linked with using less tentative (e.g., “maybe”) and differentiation (e.g., “although”) words [18] and increases confidence and leads to overconfident decision-making [19–23]. For example, high-power participants were more inclined than low-power participants to place bets on their ability to answer extremely difficult questions (before seeing the actual questions) despite the low likelihood of winning [21]. In another line of research, power was linked to an illusion of control (i.e., the belief that one is able to influence outcomes that are beyond one’s control; [24]). Finally, powerful people overestimated how accurately subordinates perceived feedback [25]. The aforementioned findings suggest that power is related to biases in thinking and decision-making that could be beneficial or harmful for oneself but also for others [26]. However, to more fully understand whether power is related to cognitive biases one prominent illusion that has not yet been related to power should be studied: the IOED.

The IOED describes people’s tendency to think they understand the world in more detail, coherence, and depth than they actually do. People only become aware that they are subject to this illusion when they attempt to explain the details of a phenomenon. Rozenblit and Keil [4] found this type of illusion only with respect to causal explanatory knowledge. In their original study, participants learned how to use a 7-point scale to assess their level of understanding of certain phenomena [4]. First, participants rated their level of understanding for various items. Then, they had to provide step-by-step explanations for selected items and afterwards rate their level of understanding for these items again. There was a reduction in self-assessed understanding between preexplanation and postexplanation ratings. When they had to provide explanations, people understood that they had overestimated the depth of their understanding. This illusion was domain-specific and pertained only to causal explanatory knowledge [27], such as complex devices or natural phenomena (e.g., “How a sewing machine works”). The IOED does not pertain to facts or simple procedures (e.g., “How to bake a cake”). With these latter types of knowledge, people typically have exact assessments of how deep their understanding is because they have more experience with assessing their knowledge depth in these domains and these domains are more transparent, that is, participants know the steps leading to a satisfying response. In later research, the IOED was demonstrated with causal explanatory knowledge regarding mental disorders [28], health improvement, climate protection [29], and policy [30–32]. Moreover, the IOED was reported to also occur in children [33].

Importantly, the IOED is different from overconfidence as the IOED pertains only to a specific type of knowledge (complex causal explanations; [27]) and requires awareness, that is, a conscious change in self-assessments occurs. Overconfidence is more general because it relates to the self and not to specific knowledge domains. Further, overconfidence is typically studied by comparing participants’ confidence in answering questions with their performance [34]. A comprehensive overview of differences between the IOED and general overconfidence can be found in Table 1.

**Table 1:** Differences between overconfidence and the IOED.

Criteria	Overconfidence	IOED
Nature of bias	Overestimation regarding accuracy, performance, or likelihood of positive outcomes (pertains to abilities, knowledge, and judgments)	Overestimation of explanatory knowledge (pertains to complex concepts or systems only)
Focus of bias	Relates to various domains (but domain-specific assessments are possible)	Relates to understanding of a particular concept (e.g., natural phenomena)
Applicability	Observable in decision making, problem solving, interpersonal skills, etc.	Observable in situations in which individuals believe they have a good understanding of how a concept works
Awareness	Not necessary (often one is not aware of the bias)	Necessary (because changes in self-report constitute the IOED)
Operationalization	Comparing individuals’ self-report with actual performance	Asking individuals to explain something and comparing their pre-explanation with their post-explanation ratings
Correction mechanism	Feedback, experience, additional information	Asking individuals to provide more detailed and accurate explanations of concepts they claim to understand

### Power, construal style, and the IOED

Different reasons have been proposed for why the IOED occurs. For example, people rarely provide explanations and are consequently bad at assessing the depth of their knowledge. Further, explanations have indeterminate end states [4] and people confuse knowledge that others provide with their own [35,36]. Alter et al. [30] conducted five experiments and reported that an abstract construal style led to the IOED. Abstract information processing comprises a focus on core aspects of information and the necessity to extract the gist (i.e., the deeper meaning) of an object or issue. Abstract construals are broad, general, and superordinate, whereas concrete construals focus on specific features [37].

Another line of research demonstrated that power is associated with abstract information processing [38–40] and abstract language [41], which makes sense because leaders need to see the big picture and take long-term goals into perspective [42]. A central element of the social distance theory of power is that power leads to abstract information processing [16]. An abstract construal style, a focus on higher order goals, and long-term desires are regarded as necessary and positive consequences of power: “After all, it seems most logical and profitable to make decisions that are driven by goals and values rather than by the small details” ([40], p. 593).

We linked the literature on power and construal style [40] with the literature on construal style and the IOED [30] and expected that there may be a dark side of abstract thinking: Power may increase the IOED. In other words, we expected a larger IOED (i.e., a larger difference between preexplanation and postexplanation ratings) for high-power participants as compared with low-power participants. (We did not make specific predictions whether a larger IOED would be driven by increased preexplanation or decreased postexplanation ratings but based on the literature the former can be considered the relevant component). According to the theorizing above, we expected abstract information processing to be a mediator: Power should increase abstract thinking and abstract thinking should go along with a stronger IOED. So far, construal style has not been considered as a mediator in the effects of power and thus the present research may provide evidence for distal predictions derived from the social distance theory of power, that is, construal style as consequence of power explains how power holders ultimately think. Moreover, this research will contribute to the ongoing debate whether power has positive or negative effects in actors [42,43].

### Narcissism as a potential moderator in the power-IOED link

Furthermore, interindividual differences can moderate effects of power on outcomes. In fact, previous research has often identified predispositions and values to determine whether power has prosocial or antisocial consequences [9,44,45]. We considered it relevant to test whether narcissism would be a potential moderating factor in the power-IOED link. It thus seems likely that people who tend towards overconfidence (i.e., narcissists) would become even more overconfident when they have power.

Narcissism as a dimensional personality trait is characterized by entitlement, feelings of grandiosity and self-importance [46]. Further, individuals with narcissistic traits often strive for attention, superiority, and typically have an exaggerated perception of their abilities and achievements [47]. Grandiose narcissism is positively related to inflated self-esteem, aggression, a need for power, extraversion, and disagreeableness [48,49].

We considered narcissism as a relevant moderator because narcissism is associated with self-enhancement [50,51], overconfidence [2], and cognitive biases such as the Dunning-Kruger effect [52,53]. Narcissists think they are more intelligent than they actually are [54] and are thus more prone than others to illusory thinking [55,56]. In fact, some researchers describe illusory biases as a key characteristic of narcissism [57].

Power typically leads to trait-behavior-correspondence and thus increases in the expression of the authentic self [58]. Thus, it can be assumed that a boost of power in narcissists increases their tendency to overestimate their own knowledge. In line with that reasoning, researchers suggested to study effects of power and narcissism together [21] and it was shown that power and narcissism can increase overconfidence [59]. Whereas the IOED is distinct from general overconfidence, we expect that the Power x Narcissism interaction may generalize across illusions and biases and thus expect higher degrees of narcissism to augment the power-IOED link.

### Overview

We conducted three studies in which participants completed the IOED paradigm. Additionally, participants’ explanations were rated by judges to provide an additional measure of the IOED—we call it judge-rated IOED. Consequently, we were able to contrast the IOED, a subjective change in understanding, with a rather objective indicator. Note that we use the term IOED also with the analysis of the observer ratings but actually this new measure may be considered a variant of the IOED as it reflects how well participants estimates are calibrated in relation to an other-rated criterion. We consider it useful to add this measure to our design to understand whether participants’ ratings actually diverge from ratings by independent observers. To ensure a potent induction of power, we pretested the power manipulations (see Online Supplement).

In Study 1, we compared the effect of power on the IOED with the effect of power on overconfidence. In Studies 2 and 3, we tested construal style as a mediator and narcissism as a moderator of the power-IOED link. Power was manipulated (Studies 1 and 2) or assessed with an established scale aimed at measuring general power feelings (Study 3). Finally, we conducted a mini meta-analysis on our experiments and checked the evidential value of previous studies on the mediating process. We report all manipulations, measures, and exclusions in all the studies we conducted (https://osf.io/p5kw3). We obtained informed consent from all participants in all studies in written form. All studies were approved by the ethics committee of the University of Bamberg (dossier number 2019-04/15). Studies were conducted between 2019 (2^nd^ December) and 2021 (5^th^ November).

## Pilot Study 1

To ensure a strong induction of power and to circumvent criticism regarding some previously used power manipulations [8], we created new manipulations that were aimed to closely fit our target population. (We also pretested a scrambled sentence task but found no difference in power feelings between high- (*M* = 4.46, *SD* = 1.19) and low-power participants (*M* = 4.57, *SD* = 0.98), *t*(68) = -.533, *p* = .666, *d* = -0.102. Thus, this manipulation was not used in the studies.) Instead, we used scenarios to manipulate power (https://aspredicted.org/blind.php?x=sr6vd8) because they have been used successfully in power research [42,60]. In the high-power condition, participants were asked to imagine that they lived in a large apartment and had received applications from potential flatmates. They were able to choose between applicants and formulated requirements for these people. Thus, high-power participants had resource and outcome control [6]—factors that have been shown to evoke a sense of power [8]. Participants in the low-power condition imagined that they had applied for a room in an unattractive apartment and urgently needed a room. Thus, they lacked resources. Then, participants completed 20 state feelings of which 16 were filler items to avoid potential demand effects. As a manipulation check, we administered the adjectives “dominant,” “inferior,” “in charge,” and “powerless” to create a score for power feelings. Afterwards, participants completed control items regarding their immersion into and identification with their role in the scenario, motivation to do the task, and empathy with their role on a 7-point scale. In total, 57 participants completed the pretest (46 women; *M*_age_ = 24.63, *SD* = 2.26, 21 to 29).

Participants in the high-power condition (*M* = 4.80, *SD* = 0.90) reported significantly higher feelings of power than participants in the low-power condition (*M* = 3.54, *SD* = 1.11), *t*(55) = 4.679, *p* < .001, *d* = 1.245. After excluding participants who had a mean that was below 4 on the aggregated control variables (i.e., the participants indicated low motivation or effort when completing the scenarios) as preregistered, the difference increased (high power: *M* = 5.03, *SD* = 0.85; low power: *M* = 3.39, *SD* = 1.04, *t*(45) = 5.862, *p* < .001, *d* = 1.715. Apparently, the task induced a large effect on experienced power and was thus used in the following main experiment.

## Study 1

In the first experiment, we aimed to analyze the direct effect of power on the IOED and additionally compared this effect with the effect of power on general overconfidence. To address the latter question, knowledge type (how devices work as examples of causal explanations vs. procedures) was designed as a between-subjects factor. Rozenblit and Keil [4] emphasized that the IOED is a cognitive illusion that occurs only with complex causal patterns (in other words, explanatory knowledge), whereas overconfidence is a phenomenon that occurs across knowledge domains. Further, empirical findings [4,33] and theoretical considerations [27] have provided evidence that the IOED and general overconfidence are different constructs. For example, the IOED is defined as a change in self-assessments, whereas overconfidence is typically apparent when comparing participants’ performance with an objective criterion. Thus, an overestimation of knowledge of procedures compared with a criterion (here judges’ ratings) reflects overconfidence, whereas an overestimation of causal knowledge (e.g., with devices) reflects the IOED. Further, procedural knowledge differs from explanatory knowledge in significant ways: The end states of procedural knowledge are typically clear, but the end state of an explanation is usually unknown. People more often engage in procedures than in providing explanations. Finally, the likelihood of encountering confusion between function and mechanism (as is the case with explanatory knowledge) is reduced in procedural knowledge. This is the case because procedural knowledge only involves understanding a sequence of highest-level functional units required to successfully accomplish a task. Accordingly, the IOED was demonstrated with explanatory knowledge only and not with procedural knowledge [4]. Thus, other-ratings of a participant’s procedural knowledge can be used as an operationalization of overconfidence that is distinct from the IOED.

We aimed to provide evidence about the question of whether power specifically promotes the IOED or general overconfidence or both. On the basis of prior findings [21], we expected to find positive effects of power on both the IOED and general overconfidence. To provide a valid measure of overconfidence and additionally to add another measure of overestimation regarding explanatory knowledge, judges rated participants’ explanations. This enabled us to contrast results from self-report and observer ratings.

### Method

#### Participants and design

We determined our sample size a priori using G*Power on information from studies on the IOED [30] and power and overconfidence [21] to obtain a valid effect size estimate (see https://aspredicted.org/blind.php?x=qj2cr5). The required sample size was 62 for between-subjects effects (ANOVA, α = .05, 1-β = .80, f = .36; for details see Online Supplement). We targeted a more conservative sample size because we wanted to be able to detect even small to medium effects and to have enough participants for analyses after exclusions. Thus, we intended to recruit at least 120 participants. Participants were recruited via email lists and through university courses. In total, 164 participants participated. One person was excluded due to language problems. The final sample comprised 163 individuals, mostly (97%) university students (85% women; *M*_*age*_ = 23.19, *SD* = 7.45, 17 to 64). As an incentive, participants were offered course credit or financial compensation (5€).

We used a 2 (between-subjects factor power: high vs. low) x 2 (between-subjects factor knowledge domain: devices vs. procedures) x 2 (within-subjects factor measurement time: t1 vs. t2 [self-report] or explanation [observer rating]) design. The measurement time factor refers to (a) the comparison of participants’ self-ratings only and (b) the comparison of participants’ self-ratings at t1 with judges’ ratings. For the latter, five judges read the explanations provided by the participants and rated participants’ level of understanding on the same scale as the participants did (see IOED Task). The judges were undergraduate students who had been trained to score explanations on the 7-point scale. Judges did not know the participants. Detailed results of how power affects the various components of the IOED, that is, preexplanation ratings, judges’ ratings, and postexplanation ratings, can be found in the Online Supplement.

#### Procedure

Participants were told that they would take part in a study concerning social and cognitive abilities. After providing informed consent, participants completed questions about demographics and read the instructions for the IOED task. Then, power was manipulated, and the manipulation check items followed. Then, participants assessed their understanding of 19 items, provided explanations for three items, and reassessed their level of understanding of these items. Finally, control items were answered. The experiment took approximately 1 hr to complete.

*Power Manipulation*. We used the flat-sharing scenario that was validated in the pilot study. Participants were randomly assigned to the high- (select a flatmate) or low-power (apply for a room in an unattractive apartment) group. The manipulation check consisted of the pretest items (“dominant,” “inferior,” “in charge,” “powerless”) that were presented along with eight filler items (emotion words) to distract participants from the experimental hypotheses. Agreement with the items was rated on a scale ranging from 1 (*not at all*) to 7 (*extremely*).

*IOED Task*. First, participants learned how to use a 7-point scale to indicate their level of understanding. Participants were instructed to choose the 1 on the scale if they knew nothing or nearly nothing about how a device/procedure works, the 4 if they assessed their knowledge as moderate, and the 7 if they thought they knew (nearly) everything about the procedure/device. After two sample items, two control items were implemented to assess whether participants correctly understood the instructions. The standard IOED task followed later [4]. Participants rated how well they thought they understood 19 phenomena (t1). Then, they were asked to explain three of these phenomena in written form and with supporting drawings if desired. After each item, they assessed how well they thought they understood the item (t2). The test items for devices were zipper, sewing machine, and tachometer. The items for procedures were how to tie a tie, how to drive from one city (Bamberg) to another (Nuremberg), and how to cook pasta.

*Control Items*. For the power manipulation, we used the same control items as in the pilot study. For the IOED task, we asked how motivated participants were to provide good explanations, how much effort they put into the task, and how seriously they had worked on the task. Responses were given on a scale ranging from 1 (*not at all*) to 7 (*extremely*).

### Results

#### Manipulation check

A one-way ANOVA with group as the between-subjects factor and feelings of power as the dependent variable was significant, *F*(3, 157) = 45.287, *p* < .001, η_p_^2^ = .464. Post hoc Scheffé tests showed that participants in the high-power groups did not differ significantly from each other (devices: *M* = 5.32, *SD* = 0.86; procedures: *M* = 5.34, *SD* = 0.85; *p* = 1.00), and neither did participants in the low-power groups (*M* = 3.65, *SD* = 1.17; *M* = 3.23, *SD* = 1.22; *p* = .365). However, and most importantly, participants in the high-power groups reported higher feelings of power than participants in the low-power groups did (all *p*s < .001, *d*s ≥ 1.613).

#### Main analyses

**Fig 1:**
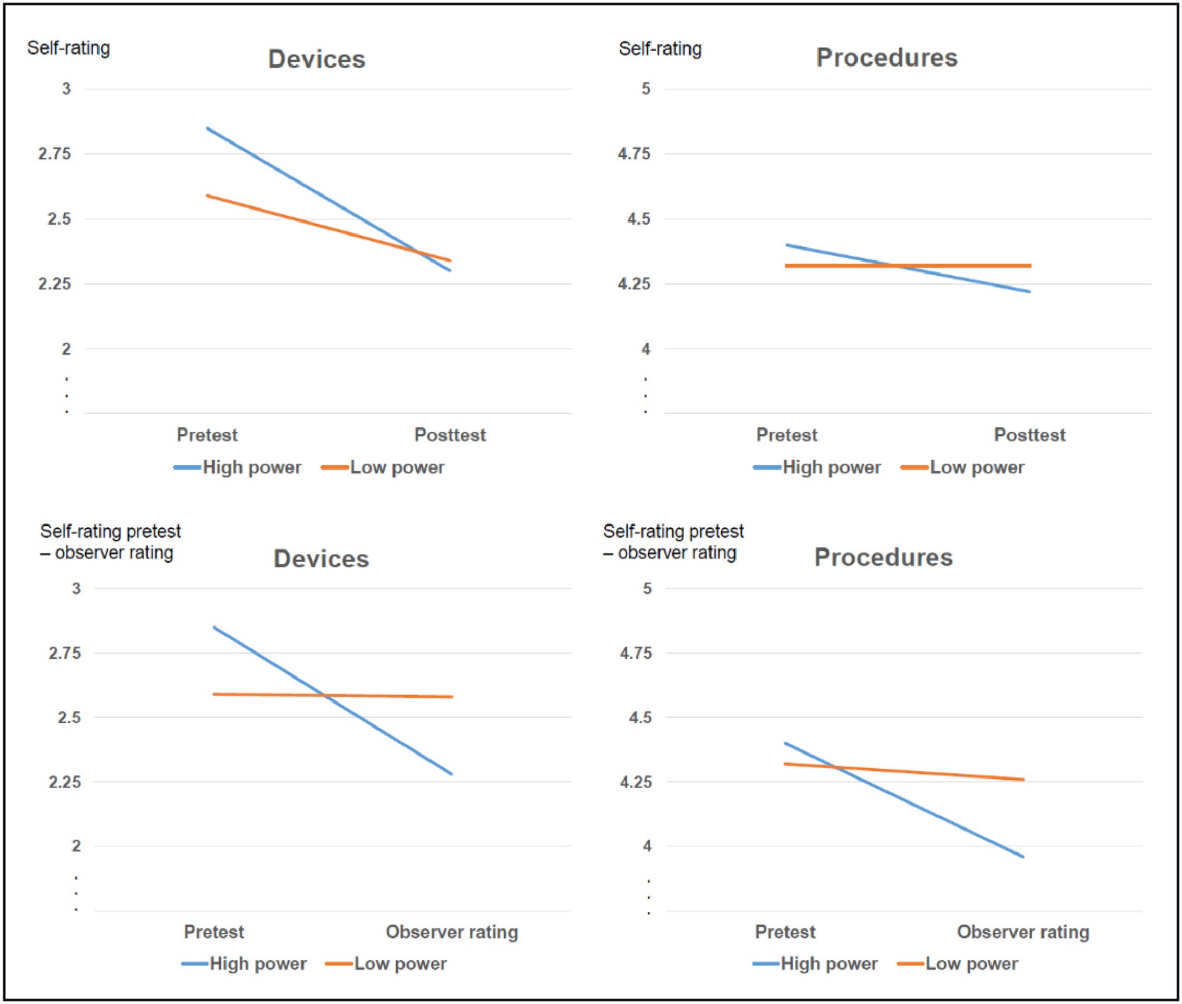
Results of Study 1 separated by knowledge domain (devices vs. procedures) and ratings (self-ratings from t1 and t2 vs. self-ratings from t1 and observer ratings).

*Self-Ratings*. Descriptive statistics across all condition are displayed in Table 2 (see also Fig 1). A three-way repeated-measures ANOVA showed a main effect of knowledge domain, *F*(1, 159) = 179.609, *p* < .001, η_p_^2^ = .530, meaning that the overall level of understanding was higher for procedures than for devices. There was also a main effect of time, *F*(1, 159) = 17.804, *p* < .001, η_p_^2^ = .101, indicating that knowledge assessment at t1 were higher than at t2. The main effects were qualified by a Power x Time interaction, *F*(1, 159) = 6.962, *p* = .009, η_p_^2^ = .042, suggesting that ratings in the high-power groups decreased to a larger extent from t1 to t2 than ratings in the low-power groups. Finally, there was also a Knowledge domain x Time interaction, *F*(1, 159) = 6.559, *p* = .011, η_p_^2^ = .040. Participants’ self-rated understanding about devices decreased more than their self-rated understanding about procedures. The three-way interaction was nonsignificant. Thus, power did not lead to a higher overestimation for devices than for procedures.

**Table 2:** Descriptive statistics for the four groups in Experiment 1.

	Before exclusion (*N* = 163)			After exclusion (*N* = 135)		
Condition	Pretest	Posttest	Observer Rating	Pretest	Posttest	Observer rating
HD	2.61 (1.05)	2.10 (0.96)	2.14 (0.60)	2.85 (1.07)	2.30 (0.98)	2.28 (0.62)
LD	2.53 (0.94)	2.33 (0.91)	2.53 (0.73)	2.59 (0.93)	2.34 (0.89)	2.58 (0.75)
HP	4.43 (0.89)	4.22 (0.98)	3.88 (1.10)	4.40 (0.94)	4.24 (1.03)	3.96 (1.01)
LP	4.24 (1.05)	4.28 (0.93)	4.20 (0.95)	4.32 (0.99)	4.32 (0.90)	4.26 (0.96)

*Note*. HD = High-power devices, LD = Low-power devices, HP = High-power procedures, LP = Low-power procedures.

Remember we expected to find a larger IOED for high-power than for low-power participants. When analyzing types of knowledge separately, we found that the IOED (devices) was present for participants in both power groups (main effect of time), *F*(1, 80) = 22.717, *p* < .001, η_p_^2^ = .221, and was indeed stronger for high- than low-power participants: The first showed a greater decrease in self-ratings than the latter, *F*(1, 80) = 4.398, *p* = .039, η_p_^2^ = .052 (Power x Time interaction). There was no support for a change in self-assessments for procedures as neither the main effect nor the interaction were significant (*p*s ≥.107), which provides support for the domain-specificity of the IOED.

When excluding participants who scored 4 or lower on the control items (*N* = 27, see the preregistration), the results of the three-way ANOVA remained similar (see the Online Supplement for detailed results). The two-way ANOVA for devices showed evidence of the IOED, *F*(1, 63) = 23.369, *p* < .001, η_p_^2^ = .271, but the Power x Time interaction missed the conventional level of significance, *F*(1, 63) = 3.414, *p* = .069, η_p_^2^ = .051. The two-way ANOVA for procedures again showed no significant main effect of time and no significant interaction (*p*s = .305).

*Observer Ratings*. We first assessed interrater agreement using the intraclass correlation. The raters showed high consensus in their assessments of procedures, *M*(ICC[2, 1/5]) = .87/.97, and devices, *M*(ICC[2, 1/5]) = .57/.86. Please note that the analyses concerning the observer ratings were exploratory and not preregistered.

As with the self-ratings, a three-way repeated-measures ANOVA showed a main effect of knowledge domain, *F*(1, 159) = 188.608, *p* < .001, η_p_^2^ = .543, and a main effect of time, *F*(1, 159) = 13.462, *p* < .001, η_p_^2^ = .078. In other words, level of understanding was higher for procedures compared with devices and higher for the self-rating at t1 compared with the judges’ ratings. Only the Power x Time interaction was significant, *F*(1, 159) = 11.275, *p* < .001, η_p_^2^ = .066; the scores in the low-power group did not differ between t1 and the judges’ ratings, but for the high-power group, the judges’ ratings were lower than the self-ratings (see Table 2).

Next, we analyzed the types of knowledge separately. For devices, there was a main effect of time, *F*(1, 80) = 5.855, *p* = .018, η_p_^2^ = .068, indicating the presence of the IOED. This main effect was qualified by a Power x Time interaction, *F*(1, 80) = 5.855, *p* = .018, η_p_^2^ = .068. The IOED was stronger for participants in the high-power group and absent for participants in the low-power group.

The focal test of whether power leads to overconfidence is the Power x Time interaction with procedures as knowledge type. There was a significant main effect of time, *F*(1, 79) = 7.591, *p* = .007, η_p_^2^ = .088, qualified by the Power x Time interaction, *F*(1, 79) = 5.467, *p* = .022, η_p_^2^ = .065. Participants in the high-power group showed a larger discrepancy between their self-ratings at t1 and the judges’ ratings than participants in the low-power group. Thus, high-power participants experienced overconfidence compared with low-power participants who hardly ever showed overconfidence. Again, the results of the two- and three-way ANOVAs after removing participants who scored 4 or lower on the control items remained similar (see the Online Supplement).

### Discussion

First, the findings demonstrated that the IOED was present for all participants. The IOED was also distinct from overconfidence—an overestimation for procedures was only found when considering observer ratings but not when comparing self-ratings across measurement times. The IOED occurred for self-ratings but also with observer ratings: when comparing self-ratings at t1 with self-ratings at t2 and when comparing self-ratings at t1 with observer ratings.

Second and more importantly, we found initial evidence that power affected both the IOED and overconfidence. With respect to the comparison of self-reports across the two measurement times, results suggested that power slightly increased the IOED. With respect to the comparison of self-reports with observer ratings, we again found that overestimation of explanatory knowledge was more pronounced in the high-power group than in the low-power group. Self-assessments of knowledge depth of procedures were not affected by power. In addition, we found that high-power participants showed overconfidence, whereas low-power participants did not. This result dovetails with the nonsignificant effect of power on knowledge depth for self-rated procedures because overconfidence can only be assessed on the basis of an objective criterion or ratings by independent observers [61]. As the effect of power on the IOED was small, we aimed to replicate this finding.

## Pilot Study 2

We pretested the effectiveness of the power manipulation for Experiment 2. Again, we used scenarios to which participants were randomly assigned but changed the content to allow for higher generalizability of the results. Participants in the high-power condition were asked to imagine that they were in a leading position of a student consultancy. They received applications from potential employees and had to decide which applicants they wanted to invite for a job interview. Then, they generated questions for the interview. Participants in the low-power condition imagined that they had applied for a job at the consultancy. They were told that they very much needed this job to pay for their expenses and were instructed to write a letter of application. The same manipulation check and filler items as in Pilot Study 1 followed. Fifty-two participants finished the pretest. Seven participants had to be excluded because they did not complete the power manipulation (22 women; *M*_age_ = 30.13, *SD* = 12.22, 19 to 68). Participants in the high-power condition (*M* = 4.78, *SD* = 1.38) reported significantly higher feelings of power than those in the low-power condition (*M* = 2.73, *SD* = 1.15), *t*(43) = 5.399, *p* < .001, *d* = 1.613. Thus, this scenario task also produced a large effect on experienced power and was thus used in the following main experiment.

## Study 2

In this study, we examined the effect of power on the IOED and tested construal style as a mediator of this relation [30,40]. As much research has shown a positive effect of power on abstract information processing [16], we expected that high power would lead to a more abstract construal style than low power and in turn to a higher IOED [30]. Additionally, we tested whether narcissism would magnify the link between power and the IOED. As in Study 2, judges rated participants’ explanations to provide evidence of whether power affects overestimation of explanatory knowledge if additionally measured with ratings of independent observers.

### Method

#### Participants and design

We aimed to gather a sample of 200 participants to have sufficient power to test for mediation via bootstrapping and recruited slightly more individuals to have enough power after exclusions (https://aspredicted.org/blind.php?x=qz782i). Participants were recruited via email lists, social media, and on campus. In total, 208 individuals took part. We excluded four participants because they did not complete the IOED or scenario task and two participants because they had implausible values on the control items for the IOED instructions, which suggested that they did not understand the instructions (i.e., ticking a 1 when an explanation was very exhaustive). The final sample consisted of 202 individuals (98% university students; 75% women; *M*_*age*_ = 22.50, *SD* = 4.30, 18 to 54). We offered course credit and 10 x 10€ Amazon vouchers for those who performed best on the tasks to ensure careful responding. There was one between-subjects factor (power: high vs. low) and one within-subjects factor (measurement time: t1 vs. t2 [self-report]/explanation [observer rating]). Again, five trained judges rated participants’ level of understanding from participants’ written explanations.

#### Procedure

The procedure was identical to Study 1 except that two scales were added (narcissism, construal style), a different power manipulation was used, and two instead of three devices were used in the IOED task to keep the experimental time identical to that of Study 1. Participants were told that the study was on social and cognitive abilities. Participants completed questions on demographics and a narcissism questionnaire. Next, they read the instructions for the IOED task. The power manipulation followed (see Pilot Study 2) for which participants were randomly assigned to the high (being in a leading position and choosing between applicants) or the low-power (applying for a job) group. Then, they completed a measure of construal style. Following that, they completed the IOED task (see Study 1). Test items were how a zipper works and how a toilet flushes. Finally, control items were given. The experiment took approximately 1 hr.

#### Measures

To measure construal style, we used the *Behavior Identification Form* [62]. Participants were informed that behaviors can be identified in different ways. Then they chose one of two alternatives for certain behavior (25 items; e.g., “making a list: (a) getting organized vs. (b) writing things down” representing (a) a high-level construal or (b) a low-level construal). We used state instructions when presenting the items. Cronbach’s alpha was good (α = .80).

The short form of the *Narcissistic Admiration and Rivalry Questionnaire* (NARQ; [63]) was used to assess narcissism. The scale captures the facets Admiration (three items; e.g., “Being a very special person gives me a lot of strength”) and Rivalry (three items; e.g., “I want my rivals to fail”). Responses were given on a scale ranging from 1 (*strongly disagree*) to 6 (*strongly agree*). Cronbach’s α was.72.

### Results

#### Manipulation check

Participants in the high-power group (*M* = 4.65, *SD* = 1.13) reported significantly higher power feelings than those in the low-power group (*M* = 2.32, *SD* = 0.89), *t*(200) = 15.890, *p* < .001, *d* = 2.254. Thus, the manipulation was effective.

#### Main analyses

*Self-Ratings*. We conducted some preliminary analyses: Narcissism did not differ between groups (*p* = .597), suggesting that the variable could be used as a moderator. However, construal style did not differ between the groups either (high power: *M* = 14.46, *SD* = 4.61; low power: *M* = 14.25, *SD* = 4.70), *t*(200) = 0.315, *p* = .753, indicating that it might not be an appropriate mediator. Further, construal style was not significantly correlated with the difference score for the IOED (t1-t2), *r*(200) = -.07, *p* = .320, which is why a mediation through construal style would be implausible.

Table 3 presents descriptive statistics for the IOED scores. When computing a repeated-measures ANOVA with self-ratings as the within-subjects factor and power as the between-subjects factor, we found evidence of the IOED because the main effect of time was significant, *F*(1, 200) = 13.217, *p* < .001, η_p_^2^ = .062. Self-ratings at t2 (*M* = 3.13, *SD* = 1.32) were significantly lower than self-ratings at t1 (*M* = 3.38, *SD* = 1.32). However, neither the main effect of power (*p* = .696) nor the interaction (*p* = .436) were significant.

**Table 3:** Descriptive statistics for Experiment 2.

	Before exclusion (*N* = 202)			After exclusion (*N* = 136)		
Condition	Pretest	Posttest	Observer Rating	Pretest	Posttest	Observer rating
High power	3.33 (1.33)	3.13 (1.39)	3.41 (1.19)	3.49 (1.45)	3.30 (1.50)	3.45 (1.15)
Low power	3.45 (1.30)	3.14 (1.22)	3.54 (0.91)	3.38 (1.11)	3.14 (1.23)	3.57 (0.96)

Using Model 5 in PROCESS, we conducted a moderated mediation analysis with power (1 = high power, 2 = low power) as the predictor, construal style as the mediator, narcissism as the moderator, and the IOED (t1-t2) as the outcome. One-tailed *p*-values are reported in the following because the model allowed us to test all of our one-tailed hypotheses. The results mirrored those from the preliminary analyses: There were no significant effects of power on construal style or the IOED, nor was there a mediation by construal style. The Power x Narcissism interaction was also not significant (see the Online Supplement).

When we excluded participants who scored below the theoretical midpoint of the control scale, the results did not change much (see the Online Supplement). Again, there was no difference in construal style between the power groups (high power: *M* = 14.82, *SD* = 4.63; low power: *M* = 14.03, *SD* = 4.65), *t*(132) = 0.978, *p* = .330. Evidence of the IOED was found (main effect of time), *F*(1, 134) = 6.466, *p* = .012, η_p_^2^ = .046, but neither the main effect of power (*p* = .541) nor the interaction (*p* = .803) were significant. Construal style was not related to the IOED, *r*(134) = -.02, *p* = .816, and the moderated mediation model showed no significant direct or indirect effects. Yet, narcissism moderated the power-IOED link (*p* = .047, one-tailed), indicating that for high-power participants, the IOED was more pronounced the higher the participant’s narcissism score was. Note that the lower limit of the 95% CI was negative [-0.07], and the result should thus be interpreted with caution.

*Observer Ratings*. The raters showed high consensus in their assessments, *M*(ICC[2, 1/5]) = .85/.97. Construal style was only weakly correlated with the IOED (t1-observer rating), *r*(200) = .109, *p* = .124. When computing a repeated-measures ANOVA with power as the between-subjects factor and measurement time as the within-subjects factor, no significant main effects nor a significant interaction were found (*p*s >.314; see Table 3 for descriptive statistics).

Results of a moderated mediation analysis showed no significant effects. When excluding participants who scored below 4 on the control items, we found weak evidence of an effect of construal style on the IOED, *b* = 0.05, *p* = .042, one-tailed, 95% CI [-0.01, 0.11]. The Power x Narcissism interaction was close to the conventional level of significance (*p* = .063, one-tailed). No other effect was significant.

### Discussion

As in Study 1, we found support for the IOED. The effect pertained only to the IOED based on self-ratings. We did not test for an IOED based on observer-ratings because the observer-ratings were descriptively higher than the self-ratings. In other words there was no drop from t1 (self-rating) to the observer-ratings of the explanations. However, with both types of assessment—comparing self-ratings across measurement times as the classical approach for assessing the IOED or when comparing self-ratings at t1 with observer ratings to assess participants’ degree of calibration of their own knowledge—there was no evidence of an effect of power on the IOED despite a very strong power manipulation. Further, there was no effect of power on construal style, and construal style was not reliably related to the IOED, which is why the mediation model was rejected. There was some evidence (though only one of the two tests was significant) that narcissism moderated the power-IOED link so that high power coupled with high narcissism led to the strongest IOED.

Why did we not find evidence of an effect of power on the outcomes in this study? One possibility is that power actually has no effect on the IOED because the IOED is too stable to be affected by specific states. Alternatively, our incentive might have weakened a potential effect: Participants were informed that they could win 10€ Amazon vouchers if they did very well on the experimental tasks. This was done to increase participants’ motivation. Yet, in the approach/inhibition theory of power, high power is related to attention to rewards [6], and according to the situated focus theory of power, powerful people are especially good at paying attention to goal-related information and performing on tasks that are goal-relevant [64]. Thus, high-power participants might have turned their attention toward details to do well on the tasks in order to win the vouchers and may thus have performed similarly to the low-power participants, who, in either case, should have shown more concrete information processing [40]. To test these two options, we conducted a third study.

## Study 3

In Study 1, weak evidence of an effect of power on the IOED was found, and in Study 2, no evidence was found. To tackle the question of whether power affects the IOED and to rule out the possibility that the incentive in Study 2 might have led to a change in performance and in the self-assessments of the high-power participants, we conducted a third study. In this study, we measured stable individual differences in experienced power because this strategy allowed us to increase the study’s ecological validity and generalize the findings. The methodology was very similar to Study 2; however, we assessed sense of power as a trait and changed the incentive. This allowed us to test the two alternative explanations proposed in the Discussion of Study 2. Again, we measured the IOED in a traditional way (comparing self-ratings) and in a novel way (judge-rated explanations).

### Method

#### Participants and procedure

We aimed to collect data from at least 200 participants to have enough power to test the mediation and were able to recruit 250 participants via a mailing list (https://aspredicted.org/blind.php?x=hi9h8s). We excluded eight participants because they did not seriously complete the IOED task (e.g., they wrote they were not interested in explaining how something works) or showed implausible responses on the control items for the IOED. The total sample consisted of 242 participants (71% women; *M*_*age*_ = 45.32, *SD* = 16.40, 18 to 78). Participants could win one of two 50€ Amazon vouchers for completing the study—independent of their task performance.

The cover story and procedure were identical to Study 2 except that we used a power scale instead of a power manipulation. The experiment took approximately 45 min. Again, five judges rated participants’ level of understanding of participants’ explanations.

#### Measures

As in Study 2, we used the *Behavior Identification Form* [62] and the *Narcissistic Admiration and Rivalry Questionnaire* (NARQ; [63], α = .72). Additionally, we employed the *Personal Sense of Power Scale* [5,9] to measure trait feelings of power. The scale assesses social influence and decision-making ability with six items (e.g., “My ideas and opinions are often ignored”). Responses are given on a scale ranging from 1 (*strongly disagree*) to 7 (*strongly agree*). Cronbach’s α in the present study was.85.

### Results

#### Self-ratings

There was strong evidence of the IOED, *F*(1, 240) = 45.681, *p* < .001, η_p_^2^ = .159, as knowledge assessments decreased from pretest (*M* = 4.22, *SD* = 1.41) to posttest (*M* = 3.82, *SD* = 1.48). Power was correlated with construal style, *r*(240) = .16, *p* = .012; however, neither power nor construal style were related to the IOED, *r*s(240) ≤ |.04|. The Power x Narcissism interaction was nonsignificant. A moderated mediation model with power as the predictor, construal style as the mediator, narcissism as the moderator, and the IOED as the outcome revealed no significant effects except for a positive link between power and construal style. When participants who scored below 4 on the control items were excluded (*N* = 41), there was again an IOED effect (η_p_^2^ = .128), and the correlations and the results of the moderated mediation model remained similar (see the Online Supplement).

#### Observer ratings

The raters showed high consensus in their assessments, *M*(ICC[2, 1/5]) = .88/.97. Again, strong evidence for the IOED based on observer ratings was found as the pretest ratings (*M* = 4.22, *SD* = 1.41) were much higher than the observer ratings (*M* = 2.92, *SD* = 1.23), *F*(1, 241) = 188.694, *p* < .001, η_p_^2^ = .439. Yet, the IOED showed only weak and non-significant correlations with power, *r*(240) = .09, *p* = .190, and construal style, *r*(240) = .06, *p* = .324. There were no significant direct, indirect, or interactive effects except for the positive link between power and construal style. When excluding participants who scored below 4 on the control items, the results remained similar: There was evidence of the IOED (η_p_^2^ = .426), but the results of the moderated mediation model did not support the hypotheses concerning construal style as a mediating factor and narcissism as a moderating factor.

### Discussion

In this final study, we examined the link between power assessed as an individual difference variable and the IOED. Whether the IOED was measured as a comparison between self-ratings or in a rather objective fashion (contrasting self-ratings with observer ratings) did not make a difference for the results. Strong support for the IOED was found. However, in line with the results of Study 2, there was no evidence of an association between experienced power and the IOED. Power was weakly correlated with abstract information processing, but neither power nor abstract information processing were significantly related to the IOED.

We also aimed to shed light on the questions of whether the IOED is too stable to be affected by power or whether power leads to a higher goal focus [64], and thus, there would not be a difference in the IOED between high- and low-power participants. Indeed, we could not rule out either of these explanations, but, as we found no effect of power on the IOED in this study, the first explanation seems plausible. Thus, it does not seem that it was solely the incentive in Study 2 that led to a nonsignificant effect of power on the IOED, but the link seems less substantial than expected.

## Meta-analytical considerations

We found a weak and significant effect in one study but nonsignificant effects of power on the IOED in the other studies. Therefore, we conducted a mini meta-analysis to estimate the overall effect in this project and have enough statistical power to detect even small effects [65]. Further, we examined the evidential value of our mediation hypotheses using *p*-curve analyses.

The meta-analyses were carried out using a fixed effects approach, that is, effect sizes were weighted by sample size. This approach is more appropriate when analyzing fewer than five studies (see the OSF for additional results using a fully random effects meta-analysis). First, all effect sizes were converted into Pearson correlations. Then, all correlations were Fisher’s *z*-transformed for analyses and back-transformed into Pearson correlations for presentation. With respect to the IOED based on self-reports, the overall effect of power on the IOED was significant but very small in size, *M*(*r*) = .07, 95% CI [-.01,.16], *Z* = 1.69, *p* = .046, and was also not significantly different from zero when we excluded participants who showed low motivation and effort (< 4 on the control items; 7-point scale), *M*(*r*) = .05, 95% CI [-.05,.14], *Z* = 1.02, *p* = .186. When computing the observer-rated IOED scores, the overall effect of power on the IOED was significantly different from zero but again weak in size, *M*(*r*) = .08, 95% CI [.00,.17], *Z* = 1.86, *p* = .032 (after exclusions: *M*(*r*) = .11, 95% CI [.01,.21], *Z* = 2.24, *p* = .013). Altogether, the meta-analytic evidence suggests that power can affect the IOED but the effect is small. By contrast, the IOED (t1-t2) was observed with an overall medium to large-sized effect, *M*(*r*) = .36, 95% CI [.28,.43], *Z* = 8.46, *p* < .001 (after exclusions: *M*(*r*) = .34, 95% CI [.25,.42], *Z* = 7.02, *p* < .001).

Next, we examined the evidential value underlying previous studies regarding the mediation model. This was done using *p*-curve analyses [66], which test the distribution of statistically significant *p*-values. Right-skewed *p*-curves indicate the presence of a true effect, whereas left-skewed *p*-curves indicate selective reporting and *p*-hacking.

First, we analyzed literature on the link between power and abstract information processing. We scanned Google Scholar for relevant articles and included eight research articles [9,38–41,67–69]. (We did not include studies that tested the reverse effect, that is, whether abstract information processing leads to heightened self-perceived or other-perceived power.) We extracted 16 relevant *p*-values and subjected them to the *p*-curve app (http://www.p-curve.com; see the OSF for our *p*-curve disclosure table and a figure of the *p*-curve). The *p*-curve was significantly right-skewed, full *p*-curve: *Z* = 4.22, *p* < .001; half *p*-curve: *Z* = 4.31, *p* < .001, and the evidential value was adequate, *Z* = 1.37, *p* = .915. Thus, there is consistent evidential value that power is positively related to abstract construal style. In this vein, in Study 3, we found a weak but significantly positive correlation between power and construal style.

Alter et al. [30] reported six studies showing that the IOED is stronger when participants adopt an abstract instead of a concrete construal style. To the best of our knowledge, there is no other research on the link between construal style and the IOED. We extracted six *p*-values of the critical tests from Alter et al. ([30]; correlation of construal style with the IOED or interaction of abstract/concrete construals with the IOED). Neither the full, *Z* = 1.76, *p* = .961, nor the half *p*-curve, *Z* = -0.38, *p* = .353, were significant, and the *p*-curves were not significantly right-skewed. Next, the observed *p*-curve was compared with the expected *p*-curves of studies with an average power of only 33%: If the *p*-curve was flatter than that of such a low-powered set of studies, evidential value would be lacking [66]. The full *p*-curve was significant, *Z* = -2.88, *p* = .002, which means that evidential value was absent. Along with the conceptual replications in our Studies 2 and 3, we concluded that construal style may not be a strong reason for why the IOED occurs. Future replication studies should tackle the link between construal style and the IOED in depth to provide conclusive evidence on the question of whether construal style is a reason for the IOED.

## General discussion

In three studies, we examined the link between power and the IOED. As power holders determine the functioning of organizations, it is important to understand whether (or not) these people are exceedingly illusory with respect to causal explanatory knowledge. If this is the case, there might be serious consequences of their own decision-making and their behavior toward subordinates. Further, we tested construal style as a potential mediator and narcissism as a potential moderator of the expected power-IOED link. Overall, power (experimentally manipulated or measured as a stable trait) showed only a very small and not reliable effect on the IOED, and construal style did not mediate the link. Narcissism interacted with experimentally induced but not with trait power in predicting the IOED. How can these findings be related to past research and theory?

First, across studies, we found strong evidence for the IOED. People indicated that their explanatory knowledge was much lower after they were asked to actually provide explanations about how certain devices worked. In other words, they became aware that their knowledge was shallower than they previously believed. This finding adds to accumulating research demonstrating that the IOED exists in various fields of explanatory knowledge [4,31,32]. Further, we presented a new operationalization of the IOED based on observer ratings and consistently found that the IOED is also present in this case. We also refer to this phenomenon as IOED because we observed an overestimation of people’s knowledge depth regarding explanatory knowledge, but we stress that this operationalization extends the original definition which focused on a change in self-assessments.

Second, we found only a small effect of power on the IOED even though we validated the power manipulations with pretests, and manipulation checks ensured strong inductions of power feelings. In Study 3, we additionally used a well-established and reliable power scale to assess habitual feelings of power as a predictor [5,9]. We conclude that both trait and state power gives people only slightly illusory beliefs with respect to complex explanatory knowledge. This can have different reasons: (a) The IOED could be a relatively stable and general variable [70]. Research on the IOED so far has focused on the question of which domains this illusion occurs in and has shown that devices but not procedures are affected. Still, to date, there is hardly any evidence that inductions of certain states may evoke a stronger (or weaker) IOED. The present research is the first to consider psychological power, or more broadly speaking, a situational antecedent of the IOED, and found only a very small effect. (b) Power does not necessarily lead to misperceptions in decision-making: Power can strengthen the ability to selectively focus on information that is most relevant for specific situations and tasks at hand [45,64]. Thus, power might lead to greater sensitivity to goal-relevant situational cues [44]. In the case of the IOED, power holders might be motivated to provide accurate assessments of their knowledge depth at t1. Remember the results of Study 2, in which powerful participants might have been more inclined to thoroughly complete the IOED task to have higher chances to win vouchers. This argument runs against the notion that power makes people illusory and shows that general overconfidence (which increases with power, e.g., [22]) differs from the IOED. By combining these competing assumptions, it is also possible that the different effects of power may cancel each other out, and thus, there would not be an observable effect. (c) A third explanation is that abstract information processing as a consequence of power is not an antecedent of the IOED, which we consider most likely and discuss below.

Third, we found that the effect of power on abstract construal style was small and that construal style was not related to the IOED. Past research has investigated the effects of power on various variables [6,42] such as construal styles—but these variables have not yet been systematically studied as possible mediators in research on the downstream effects of power. We tested construal style as a mediator because there was compelling evidence regarding effects of power on this variable [16]. Nevertheless, we could not validate construal style as a mediator. Apparently, construal style is affected by power, but the assumed link between construal style and the IOED was not found. Thus, the latter seems to be the missing link in the expected indirect effect of power on the IOED. *P*-curve analyses suggested that there was not much evidential value of an effect of construal style on the IOED. It is possible that other reasons for the IOED are more relevant (e.g., confusing one’s mental representation of how something works with environmental support; [4,35]).

Fourth, we tested narcissism as a potential moderator of the power-IOED link and found only weak support for this hypothesis. Again, the IOED may be too general a tendency that does not vary much interpersonally. Yet, in line with the results of Study 2, we think that a situational boost of power in people high in narcissism might lead to a stronger IOED. Future research concerning the link between narcissism, power, and the IOED might be most promising when people who score very high in narcissism are tested—because power plus extreme narcissism could in fact be a toxic composite for the IOED (for a similar argument see [59]).

Finally, we found that power led to overconfidence as in previous research [21,22,24]. This finding provides support for the notion that power can lead to general overconfidence but that the IOED is a distinct phenomenon that does not seem to be affected by power. Typically, people are not aware of the fact that they are overly confident; however, they can become aware of their lack of explanatory knowledge when they are asked how a device works. Consequently, power seems to only slightly affect an illusion (e.g., the IOED) of which people can become aware but may affect decisions or ratings when people are unaware of their general overconfidence. In line with that, in past research power did not lead to overconfidence when participants received negative feedback [22]: The IOED task points participants to the fact that their knowledge may be shallow. By contrast, typical tasks that measure overconfidence do not include such feedback. Further, knowledge domains are important to consider because, as shown in self-ratings and in observer ratings, the IOED is a phenomenon that pertains to explanatory depth regarding devices that seem familiar at first glance even though the mechanisms are not really understood.

The present study may have implications for practice and theory. People in positions of power (e.g., managers, professors, politicians) who experience power [8] may be overconfident, but there is no evidence that such power holders tend to overestimate the depth of their causal explanatory knowledge more than others do (at least with respect to devices). In fact, to achieve a good calibration of one’s knowledge, it may be helpful for people to first provide explanations of complex issues before they provide estimates of how good their knowledge is. Yet, as powerful people do not differ from powerless people in this domain, it is not necessary to develop specific interventions targeting power holders. Apparently, starting to explain complex issues about, for example, devices, helps people to better calibrate their self-perceptions of their knowledge depth—independent of their standing in the social hierarchy. However, power in combination with toxic traits could increase the IOED. Thus narcissism and other dispositions should be more closely studied in future studies on the topic.

Further, in our research, we did not find evidence that power holders’ abstract construal style [40] has a dark side with respect to decision-making because construal style is not related to the IOED. The findings support the social distance theory of power [16] insofar as power was related to construal style. Yet, construal style was not a reason for the IOED.

Altogether, this research adds to the literature in social, personality, and cognitive psychology as well as organizational behavior in showing the presence of the IOED overall. However, the effects of power pertain largely to overconfidence and not much to the IOED. More research is needed to address the consequences of power and whether these consequences (e.g., construal style, approach behavior, positive emotion) can directly affect other downstream consequences such as decision processes or cognitive biases.

### Limitations and future research

In the present studies, we did not use a control group to contrast the effects of the two experimental groups with that group. However, as the high-power group did not largely differ from the low-power group regarding the IOED, it seems implausible that a relevant effect would have been detected with a control group. Moreover, a previous study suggested that effects of power might not necessarily be linear [71]; however, an inspection of the data from Study 3 did not show a curvilinear relation.

Our samples were similar to those of others that studied the link between power and overconfidence [21,29]. However, a limitation pertains to the overrepresentation of women in our samples. Although previous research suggests that the IOED as well as effects of power on overconfidence do not differ between sexes [21,30], in future studies, more diverse and gender-balanced samples should be used to increase generalizability. Further, our participants in Study 3 were on average more than 20 years older than the participants in Studies 1 and 2. Yet, as power is barely related to age [9] and as the results differed between Studies 1 and 2 as well as between 1 and 3 (but not between Studies 2 and 3 which have the largest age difference), it can be assumed that the age differences are not a major issue.

Further, to keep the experimental time reasonable, we did not assess construal style in Study 1. Yet, future research may benefit from testing whether construal style can mediate the power-overconfidence relation. For this purpose, different measures of abstract information processing should be employed (e.g., categorization tasks or gestalt completion tasks [40]). Upcoming studies may also benefit from conducting comparisons of extreme groups. For example, researchers could assess the IOED or other illusions in managers or subordinates or use samples with extreme narcissism scores.

We used different power operationalizations across studies. In Studies 1 and 2, we used a structural power manipulation that instilled state power in participants (consider the manipulation checks on feelings of power). In Study 3, we used a measure of trait power. Yet, the results differed mostly between Study 1, on the one hand, and Studies 2 and 3, on the other. Thus, the power operationalizations do not seem to be responsible for the inconclusive findings. Nevertheless, future studies on power and IOED should pay closer attention to the comparability of studies depending on the measure of power (e.g., trait power, state power, interviews with actual managers and subordinates, etc.).

Finally, we found no reliable support that power leads to the IOED, but we cannot rule out the possibility that power in combination with other personality or situational variables may increase the IOED. Thus, other situational factors beyond power (e.g., impression management concerns) can be investigated in relation to the IOED to examine the plausibility of the reasons we suspected for why power did not affect the IOED. Another interesting question is whether effects of power (overconfidence and illusory thinking vs. heightened sensitivity to tasks at hand) may even each other out and obfuscate effects on the IOED.

### Conclusion

The experience of power has been related to several positive consequences [6] but also to negative aspects, such as illusory thinking [21]. The present research supports the notion that power is associated with overconfidence. However, in three studies in which power was manipulated or measured, we found only a very small effect of power on the IOED. Possibly, power has stronger effects on general overconfidence than on the IOED because the processes in these two forms of illusions are different: General overconfidence probably occurs at a less conscious level than the IOED and the IOED focuses on a specific kind of knowledge which is explanatory whereas overconfidence is more general. What we have shown in any case: Power does not necessarily increase all forms of illusions. Apparently, people are subject to an IOED relatively independent of their experienced power.
